# Exploring the causal relationship between psoriasis and osteoarthritis through a 2-sample Mendelian randomization study

**DOI:** 10.1097/MD.0000000000039303

**Published:** 2024-08-16

**Authors:** Haoyu Yao, Peizhi Lu, Ya Li, Shuo Yang, Shijie Wang, Zihao Fan, Rende Ning

**Affiliations:** a Department of Orthopedics, The Third Affiliated Hospital of Anhui Medical University (The First People’s Hospital of Hefei), Hefei, Anhui, China.

**Keywords:** Mendelian randomization, osteoarthritis, psoriasis, psoriatic arthritis

## Abstract

Previous research has demonstrated a robust association between osteoarthritis (OA) and psoriasis. Notably, a significant proportion of psoriasis patients exhibit symptoms of arthritis, particularly psoriatic arthritis. However, a definitive causal relationship between psoriasis, psoriatic arthritis and OA remains to be established. This study aimed to elucidate the causal relationship between psoriasis, psoriatic arthritis, and osteoarthritis using a 2-sample Mendelian randomization approach. The causal relationship between psoriasis, psoriatic arthritis and OA was rigorously investigated using a 2-sample Mendelian Randomization (MR) approach. Instrumental variables pertinent to psoriasis, psoriatic arthritis and 4 distinct types of OA (knee osteoarthritis (KOA), hand osteoarthritis (HOA), total knee replacement (TKR), and total hip replacement (THR)) were sourced from extensive, published genome-wide association studies (GWAS). To estimate the causal effects, methodologies such as inverse variance weighting (IVW), MR-Egger, and weighted median estimation (WM) were employed. Mendelian Randomization analysis suggested a potential causal effect of psoriasis on osteoarthritis (OA). For hand OA (HOA), the *P* value was .381 (OR = 0.28); for knee OA (KOA), the *P* value was .725 (OR = 1.46); for TKR, the *P* value was .488 (OR = 0.274); and for THR, the *P* value was .454 (OR = 0.216). Furthermore, we explored the causality of psoriatic arthritis on OA. For HOA, the *P* value was .478 (OR = 0.0095); for KOA, the *P* value was .835 (OR = 0.345); for THR, the *P* value was .807 (OR = 0.120); and for TKR, the *P* value was .860 (OR = 0.190). Our findings indicate that there is no evidence of a causal connection between psoriasis or psoriatic arthritis and OA, suggesting that while psoriasis may contribute to arthritis, it does not influence OA development.

## 1. Introduction

Osteoarthritis (OA), alternatively referred to as proliferative arthritis, degenerative arthritis, or osteoarthropathy, is one of the most prevalent types of arthritis and predominantly affects the middle-aged and elderly demographics.^[1–3]^ As individuals age, there is progressive degeneration of joint cartilage, leading to joint stiffness, deformities, reduced mobility, and ultimately, disability.^[4]^ In China, the general prevalence of OA is approximately 15%, increasing with age, reaching 50% in individuals over 60 years of age and up to 80% in those over 70 years of age.^[5]^ While numerous treatments for osteoarthritis exist, the majority are symptomatic, with no established effective methods to cure or halt its progression.

Psoriasis, an immune-mediated chronic inflammatory disorder, exhibits a variable yet globally increasing incidence. Psoriatic arthritis (PsA) frequently co-occurs with psoriasis, with approximately 80% of cases presenting joint symptoms subsequent to skin manifestations,^[6]^ and If not timely diagnosed and adequately treated, PsA can result in irreversible joint damage, potentially leading to disability, markedly reducing quality of life and substantially increasing socioeconomic burdens.^[7–9]^ Initial clinical investigations have revealed a substantial correlation between OA and psoriasis, with these conditions frequently co-occurring in clinical settings; however, prior studies have not conclusively established a causal relationship between OA and psoriasis. Assessing the causal link between OA and psoriasis could unveil novel strategies for their prevention, diagnosis, and treatment.

Recent investigations have extensively utilized Mendelian randomization (MR) methods to substantiate causal relationships between various exposures and outcomes.^[10–12]^ Genome-wide association studies (GWAS) have significantly advanced the identification of single nucleotide polymorphisms (SNPs) linked to common complex diseases.^[13]^ Utilizing SNPs as instrumental variables (IVs) enables the MR approach to yield reliable causal inferences, independent of confounding postnatal lifestyle or environmental factors.^[14]^ A previous study has documented a pronounced association between osteoarthritis and psoriasis.^[15]^ Consequently, our study aims to elucidate the causal relationship between OA and psoriasis using a 2-sample Mendelian randomization approach.

## 2. Materials and methods

### 2.1. Study overview

**Figure 1.**
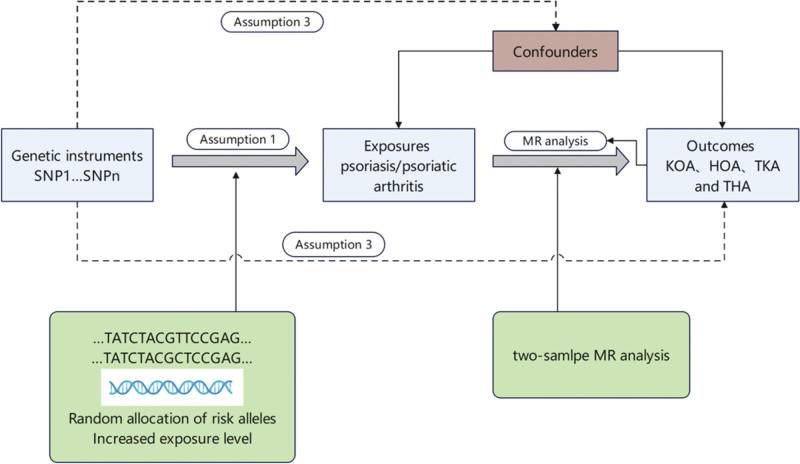
This figure delineates the 3 fundamental postulates of Mendelian randomization (MR): genetic variants are strongly associated with the exposure, evidenced by a *P* value < 5e−08; these genetic variants are not associated with known confounders; the influence of genetic variants on outcomes is exclusively mediated through the exposure.

**Figure 2.**
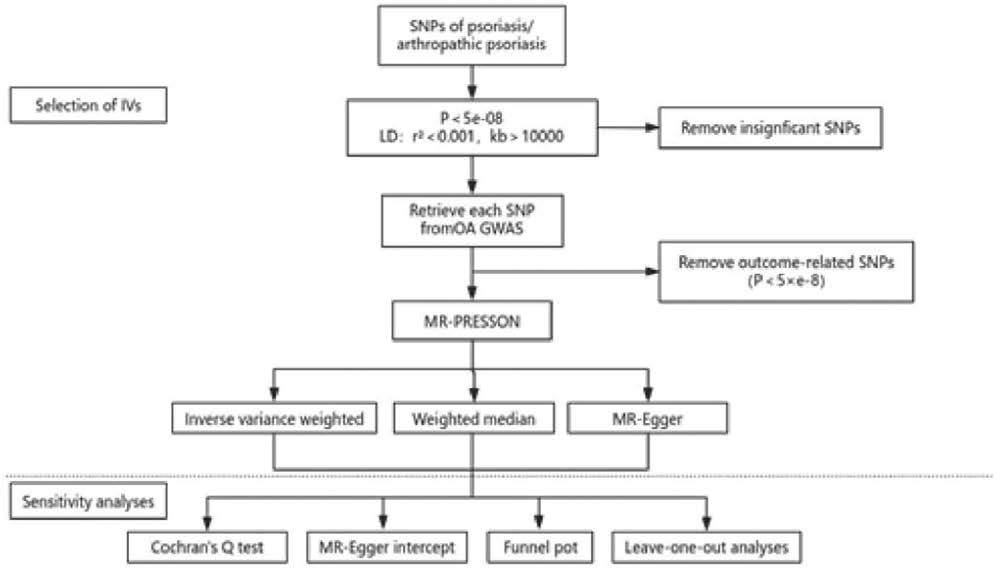
Illustration of the 2-sample Mendelian randomization (MR) analysis process.

This study utilized an existing dataset from a large-scale genome-wide association study. The causal link between psoriasis and osteoarthritis (OA) was investigated using a 2-sample MR approach, employing genetic variants of psoriasis as IVs. MR operates on 3 fundamental assumptions (as depicted in Fig. 1), which the chosen IVs must satisfy. The research flow of this study is shown in Figure 2.

### 2.2. Data sources

This study investigated 4 lower extremity osteoarthritis (OA) phenotypes: knee osteoarthritis (KOA), hand osteoarthritis (HOA), total knee replacement (TKR), and total hip replacement (THR). Summary statistics for OA were extracted from the largest genome-wide association study (GWAS) conducted by the genetics of osteoarthritis (GO) Consortium, encompassing 826,690 individuals (177,517 cases and 649,173 controls). Data for this study were sourced from cases of KOA (n = 62,497), HOA (n = 36,445), TKR (n = 18,200), THR (n = 23,021), along with a substantial number of healthy controls OA was characterized as self-reported, clinically diagnosed, ICD10 coded, or radiologically confirmed, depending on the available data within the cohort. No individuals with OA or population-based controls were included or excluded solely based on ICD codes. Genetic variants were categorized based on their specific association sites with OA. GWAS data for psoriasis (n = 361,141) and psoriatic arthritis (n = 361,194) were sourced from the UK Biobank, encompassing comprehensive data for both conditions.

### 2.3. Selection and validation of genetic instrumental variables

In alignment with the first postulate of MR, SNPs serving as IVs should exhibit a genome-wide association with the exposure; hence, we retained SNPs significant at the genome-wide level (*P* < 5e−08). Furthermore, we conducted a linkage disequilibrium (LD) analysis on the retained SNPs, applying a threshold of kb = 10,000 and *r*2 = 0. 001; SNPs failing to meet these criteria were excluded. SNPs with an *F* statistic < 10 were eliminated. The *F* statistic was calculated using the formula: *F* = [*R*2/(1 − *R*2)] × [(*N* − *K* − 1)/*K*]. Subsequently, palindromic SNPs and incompatible SNPs, where the direction could not be determined, were also excluded. Finally, all SNPs were tested for their association with the outcome, and any SNP with a *P* value < 5e−08 was removed.

### 2.4. Estimation of causal effect and sensitivity analysis

To investigate the causal relationship between psoriasis, psoriatic arthritis, and OA, a 2-sample MR analysis was conducted.

Our primary research methods included the inverse variance weighting (IVW), the weighted median (WM), and the MR-Egger method. The IVW method yields consistent estimates of the causal effects of exposure on outcomes, offering the highest statistical power, provided each instrumental variable (IV) fulfills the MR assumptions. Consequently, IVW was employed as the primary analytical method to evaluate the impact of exposure on outcomes. In addition, the WM and MR-Egger methods were utilized as supplementary analyses to enhance the reliability of the results. Cochran *Q* was applied for heterogeneity testing, Egger intercept for multivariate testing, and MR-PRESSO to identify and exclude outliers and *P* value < .05 indicative of horizontal pleiotropy.

To ensure the robustness of our findings, we conducted a leave-one-out sensitivity analysis to assess the impact of individual SNPs on the results.

Additionally, funnel plot analysis and leave-one-out analysis were conducted to further enhance the reliability of the findings.

## 3. Results

### 3.1. The analysis did not reveal a causal association of psoriasis on OA

**Figure 3.**
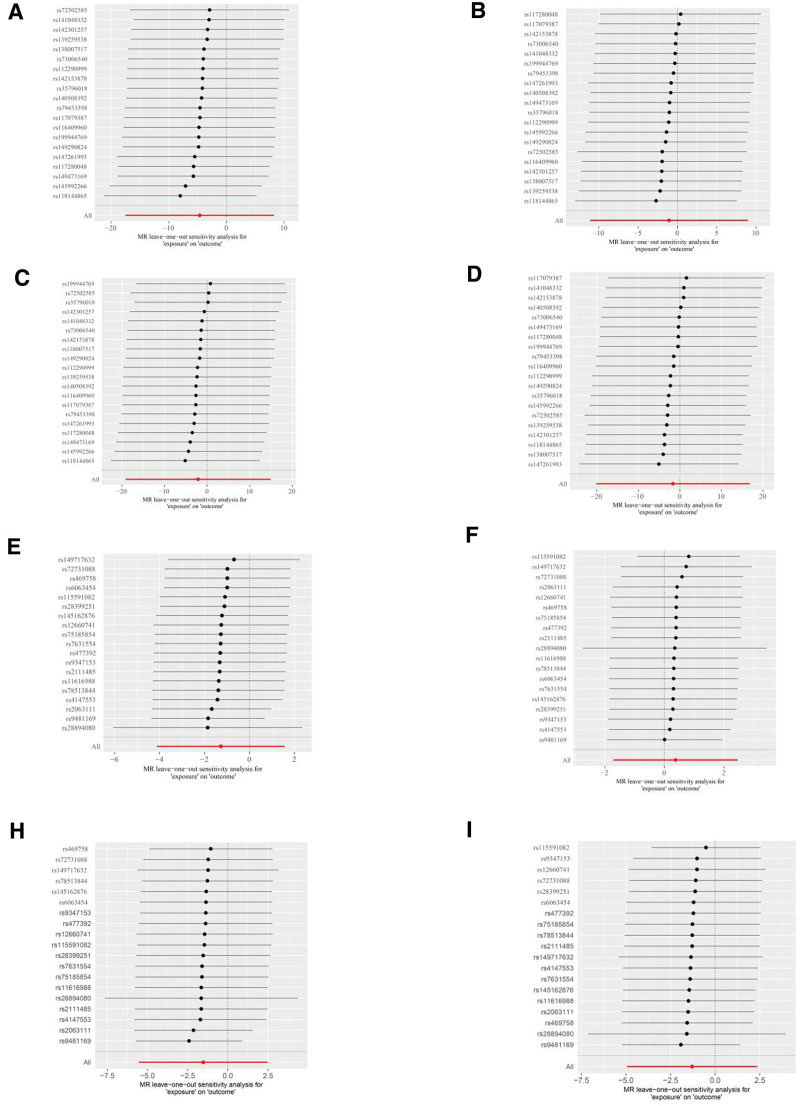
Leave-one-out analysis: (A) psoriatic arthritis-HOA; (B) psoriatic arthritis-KOA; (C) psoriatic arthritis-THR; (D) psoriatic arthritis-TKR; (E) psoriasis-HOA; (F) psoriasis-KOA; (G) psoriasis-THR; (H) psoriasis-TKR. HOA = hand osteoarthritis, KOA = knee osteoarthritis, THR = total hip replacement, TKR = total knee replacement.

**Figure 4.**
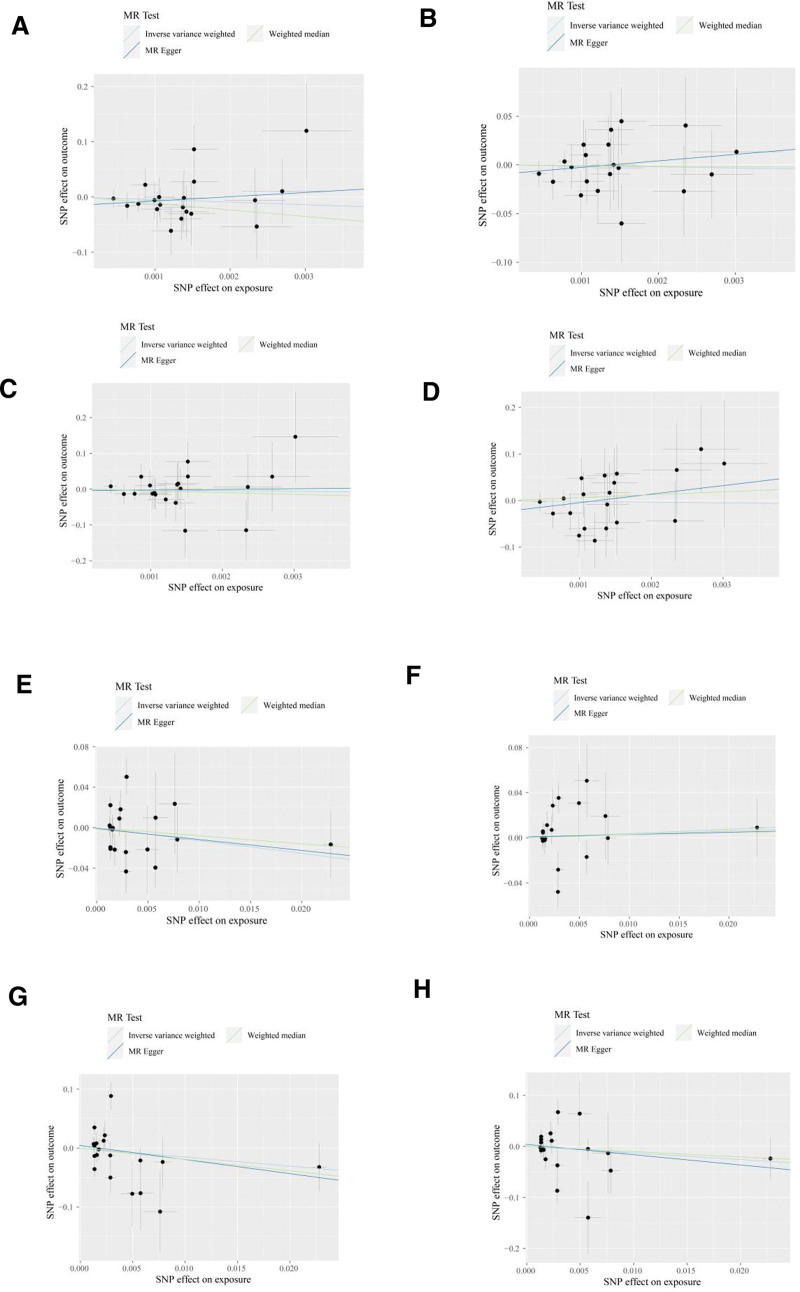
Scatterplot analysis: (A) psoriatic arthritis-HOA; (B) psoriatic arthritis-KOA; (C) psoriatic arthritis-THR; (D) psoriatic arthritis-TKR; (E) psoriasis-HOA; (F) psoriasis-KOA; (G) psoriasis-THR; (H) psoriasis-TKR. HOA = hand osteoarthritis, KOA = knee osteoarthritis, THR = total hip replacement, TKR = total knee replacement.

**Figure 5.**
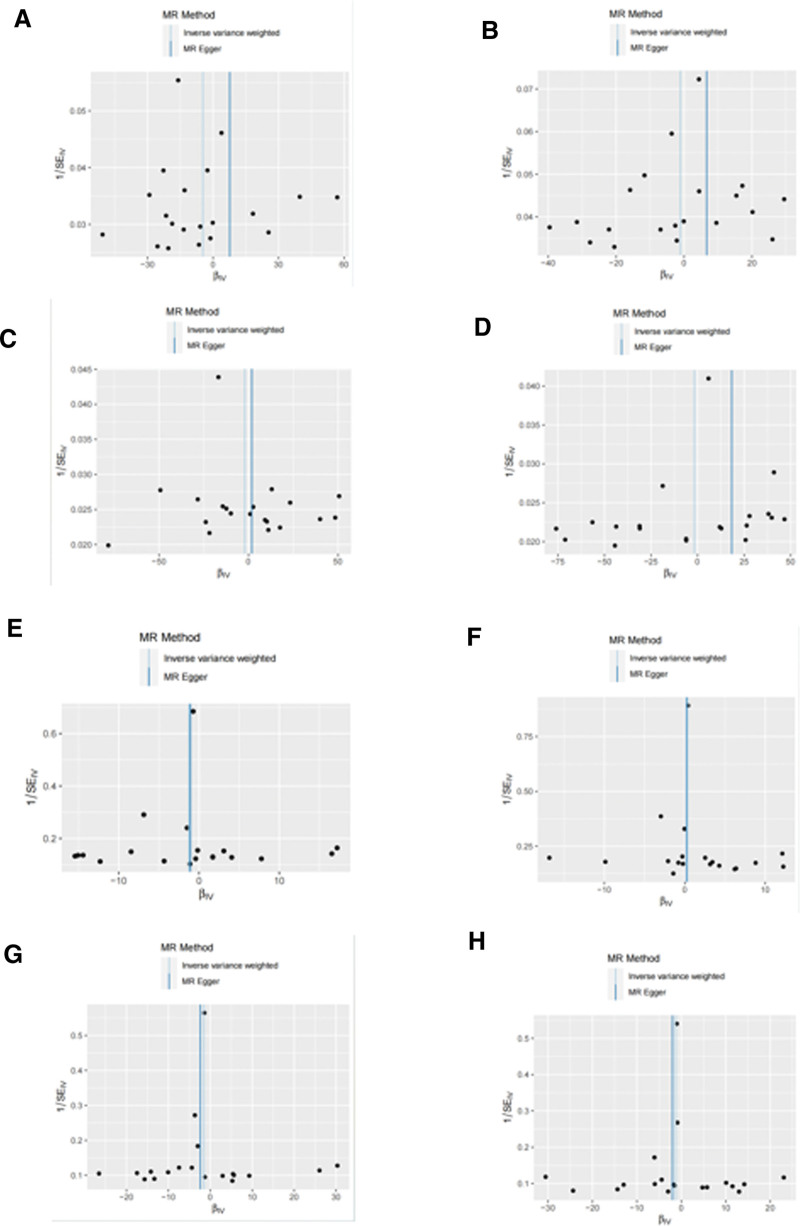
Funnel plot analysis: (A) psoriatic arthritis-HOA; (B) psoriatic arthritis-KOA; (C) psoriatic arthritis-THR; (D) psoriatic arthritis-TKR; (E) psoriasis-HOA; (F) psoriasis-KOA; (G) psoriasis-THR; (H) psoriasis-TKR. HOA = hand osteoarthritis, KOA = knee osteoarthritis, THR = total hip replacement, TKR = total knee replacement.

To assess the genetic impact of psoriasis on various outcomes, we first filtered the exposure data with a significance threshold of *P* < 5e−06. Detailed results can be found in the supplemental materials as follows: the filtered data for psoriasis exposure and hip osteoarthritis outcome are presented in Supplemental Table 1, Supplemental Digital Content, http://links.lww.com/MD/N359 (psoriasis-HOA); the filtered data for psoriasis exposure and knee osteoarthritis outcome are shown in Supplemental Table 2, Supplemental Digital Content, http://links.lww.com/MD/N359 (psoriasis-KOA); the filtered data for psoriasis exposure and total hip replacement outcome are provided in Supplemental Table 3, Supplemental Digital Content, http://links.lww.com/MD/N359 (psoriasis-THR); and the filtered data for psoriasis exposure and total knee replacement outcome are displayed in Supplemental Table 4, Supplemental Digital Content, http://links.lww.com/MD/N359 (psoriasis-TKR). Next, we pruned the data to remove linkage disequilibrium. The pruned data for psoriasis exposure and HOA outcome are presented in Supplemental Table 9, Supplemental Digital Content, http://links.lww.com/MD/N359 (psoriasis-HOA); the pruned data for psoriasis exposure and KOA outcome are shown in Supplemental Table 10, Supplemental Digital Content, http://links.lww.com/MD/N359 (psoriasis-KOA); the pruned data for psoriasis exposure and THR outcome are provided in Supplemental Table 11, Supplemental Digital Content, http://links.lww.com/MD/N359 (psoriasis-THR); and the pruned data for psoriasis exposure and TKR outcome are displayed in Supplemental Table 12, Supplemental Digital Content, http://links.lww.com/MD/N359 (psoriasis-TKR). Furthermore, we conducted an intersection analysis between the exposure and outcome data to identify genetic variants that may directly influence the outcomes. The intersected data for psoriasis exposure and HOA outcome are presented in Supplemental Table 17, Supplemental Digital Content, http://links.lww.com/MD/N359 (psoriasis-HOA); the intersected data for psoriasis exposure and KOA outcome are shown in Supplemental Table 18, Supplemental Digital Content, http://links.lww.com/MD/N359 (psoriasis-KOA); the intersected data for psoriasis exposure and THR outcome are provided in Supplemental Table 19, Supplemental Digital Content, http://links.lww.com/MD/N359 (psoriasis-THR); and the intersected data for psoriasis exposure and TKR outcome are displayed in Supplemental Table 20, Supplemental Digital Content, http://links.lww.com/MD/N359 (psoriasis-TKR). Finally, we harmonized the data across different studies to ensure consistency and comparability of our findings. The harmonized data for psoriasis exposure and HOA outcome are presented in Supplemental Table 25, Supplemental Digital Content, http://links.lww.com/MD/N359 (psoriasis-HOA); the harmonized data for psoriasis exposure and KOA outcome are shown in Supplemental Table 26, Supplemental Digital Content, http://links.lww.com/MD/N359 (psoriasis-KOA); the harmonized data for psoriasis exposure and THR outcome are provided in Supplemental Table 27, Supplemental Digital Content, http://links.lww.com/MD/N359 (psoriasis-THR); and the harmonized data for psoriasis exposure and TKR outcome are displayed in Supplemental Table 28, Supplemental Digital Content, http://links.lww.com/MD/N359 (psoriasis-TKR). Upon integrating GWAS data on psoriasis and excluding SNPs linked to outcomes or confounders, we identified 19 independent IV pairs for KOA, HOA, TKR, and THR, and conducted MR analysis. The results are presented in Table 1. The primary IVW analyses did not reveal evidence supporting a causal relationship between psoriasis and the 4 types of OA. Similarly, the MR-Egger and WM methods also yielded consistent results. In the analysis of the 4 types of OA, the MR multivariate test indicated no evidence of horizontal pleiotropy. The Cochran *Q* test results indicated heterogeneity across the results, leading us to employ a random-effects model in all cases. The leave-one-out analysis demonstrated that none of the SNPs significantly influenced the MR results, as illustrated in Figure 3. The outcomes of the scatterplot analysis are depicted in Figure 4 and the outcomes of the funnel plot analysis are depicted in Figure 5. Overall, the MR analysis results did not establish a causal relationship between psoriasis and OA.

**Table 1:** The results are detailed in this table.

Exposure	Outcome	Nsnp	Inverse variance weighted		Weighted median	MR-Egger	MR-Egger regression	
			*P* value	Heterogeneity test Cochran *Q* (p)	*P* value	*P* value	Egger intercept	*P* value
Psoriasis	HOA	19	. 381	0. 013	. 557	.591	−0. 0009379581	.883
	KOA	19	. 725	0. 025	.772	.892	0. 004642099	.855
	THR	19	. 454	0. 000347	.241	.241	0. 008952741	.640
	TKR	19	. 488	0. 0131	.527	.432	0. 008400517	.661
Psoriatic arthritis	HOA	20	. 478	0. 847	.201	.609	0. 01584202	.362
	KOA	20	. 835	0. 913	.934	.572	0. 01244065	.467
	THR	20	. 807	0. 922	.681	.929	0. 02055275	.831
	TKR	20	. 860	0. 743	.643	.405	0. 02213853	.315

Exposure: The exposure factors studied, including psoriasis and psoriatic arthritis. Outcome: The outcome variables studied, including HOA (hip osteoarthritis), KOA (knee osteoarthritis), THR (total hip replacement), and TKR (total knee replacement). Nsnp = The number of genetic variants used.

### 3.2. No causal effects evidence of psoriatic arthritis on OA

To assess the genetic impact of psoriatic arthritis on various outcomes, we first filtered the exposure data with a significance threshold of *P* < 5e−06. Detailed results can be found in the supplemental materials as follows: the filtered data for psoriatic arthritis exposure and HOA outcome are presented in Supplemental Table 5, Supplemental Digital Content, http://links.lww.com/MD/N359 (psoriatic arthritis-HOA); the filtered data for psoriatic arthritis exposure and KOA outcome are shown in Supplemental Table 6, Supplemental Digital Content, http://links.lww.com/MD/N359 (psoriatic arthritis-KOA); the filtered data for psoriatic arthritis exposure and THR outcome are provided in Supplemental Table 7, Supplemental Digital Content, http://links.lww.com/MD/N359 (psoriatic arthritis-THR); and the filtered data for psoriatic arthritis exposure and TKR outcome are displayed in Supplemental Table 8, Supplemental Digital Content, http://links.lww.com/MD/N359 (psoriatic arthritis-TKR). Next, we pruned the data to remove linkage disequilibrium. The pruned data for psoriatic arthritis exposure and HOA outcome are presented in Supplemental Table 13, Supplemental Digital Content, http://links.lww.com/MD/N359 (psoriatic arthritis-HOA); the pruned data for psoriatic arthritis exposure and KOA outcome are shown in Supplemental Table 14, Supplemental Digital Content, http://links.lww.com/MD/N359 (psoriatic arthritis-KOA); the pruned data for psoriatic arthritis exposure and THR outcome are provided in Supplemental Table 15, Supplemental Digital Content, http://links.lww.com/MD/N359 (psoriatic arthritis-THR); and the pruned data for psoriatic arthritis exposure and TKR outcome are displayed in Supplemental Table 16, Supplemental Digital Content, http://links.lww.com/MD/N359 (psoriatic arthritis-TKR). Furthermore, we conducted an intersection analysis between the exposure and outcome data to identify genetic variants that may directly influence the outcomes. The intersected data for psoriatic arthritis exposure and HOA outcome are presented in Supplemental Table 21, Supplemental Digital Content, http://links.lww.com/MD/N359 (psoriatic arthritis-HOA); the intersected data for psoriasis exposure and KOA outcome are shown in Supplemental Table 22, Supplemental Digital Content, http://links.lww.com/MD/N359 (psoriatic arthritis-KOA); the intersected data for psoriatic arthritis exposure and THR outcome are provided in Supplemental Table 23, Supplemental Digital Content, http://links.lww.com/MD/N359 (psoriatic arthritis-THR); and the intersected data for psoriatic arthritis exposure and TKR outcome are displayed in Supplemental Table 24, Supplemental Digital Content, http://links.lww.com/MD/N359 (psoriasis-TKR). Finally, we harmonized the data across different studies to ensure consistency and comparability of our findings. The harmonized data for psoriatic arthritis exposure and HOA outcome are presented in Supplemental Table 29, Supplemental Digital Content, http://links.lww.com/MD/N359 (psoriatic arthritis-HOA); the harmonized data for psoriatic arthritis exposure and KOA outcome are shown in Supplemental Table 30, Supplemental Digital Content, http://links.lww.com/MD/N359 (psoriatic arthritis-KOA); the harmonized data for psoriatic arthritis exposure and THR outcome are provided in Supplemental Table 31, Supplemental Digital Content, http://links.lww.com/MD/N359 (psoriatic arthritis-THR); and the harmonized data for psoriatic arthritis exposure and total knee replacement outcome are displayed in Supplemental Table 32, Supplemental Digital Content, http://links.lww.com/MD/N359 (psoriatic arthritis-TKR). For psoriatic arthritis, post-integration of GWAS data and exclusion of SNPs linked to outcomes or confounders, we identified 20 independent IV pairs for KOA, HOA, TKR, and THR for MR analysis. The results are detailed in Table 1. The primary IVW analyses did not reveal evidence supporting a causal relationship between psoriatic arthritis and the 4 types of OA. Similarly, the MR-Egger and WM methods also yielded consistent results. In the analysis of the 4 types of OA, the MR multivariate test indicated no evidence of horizontal pleiotropy. The Cochran *Q* test results indicated heterogeneity across the results, leading us to employ a random-effects model in all cases. The leave-one-out analysis demonstrated that no individual SNP significantly influenced the MR results, as depicted in Figure 3. The outcomes of the scatterplot analysis are depicted in Figure 4 and the outcomes of the funnel plot analysis are depicted in Figure 5. Overall, the MR analysis results did not establish a causal relationship between psoriatic arthritis and osteoarthritis (OA).

## 4. Discussion

Eighty percent of psoriasis patients typically exhibit symptoms of arthritis, and this type of arthritis presents pathological features similar to osteoarthritis, such as bone destruction and proliferation.^[16]^ Therefore, exploring the potential causal relationship between psoriasis and osteoarthritis is necessary, which holds significant clinical implications, and represents the inaugural 2-sample Mendelian Randomization analysis exploring the association between psoriasis, including psoriatic arthritis, and osteoarthritis, utilizing large-scale GWAS data. The MR analysis indicated evidence of causality was absent between psoriasis, psoriatic arthritis and OA.

### 4.1. Psoriasis, psoriatic arthritis, and osteoarthritis

Psoriatic arthritis (PsA) frequently co-occurs with psoriasis, with approximately 80% of cases exhibiting joint symptoms subsequent to skin manifestations. Without timely diagnosed and treated, PsA can result in irreversible joint damage, potentially leading to disability, markedly reducing quality of life and substantially increasing socioeconomic burdens. The question of whether psoriatic arthritis and OA are similar forms of arthritis, or if PsA can induce OA, remains unanswered. Although there are reports suggesting a link between the psoriasis and OA,^[16]^ clear evidence is still lacking. This study was conducted to explore the causal relationship between psoriasis, psoriatic arthritis, and osteoarthritis. The results did not indicate a causative association between psoriasis, psoriatic arthritis, and osteoarthritis.

### 4.2. Strengths and limitations

This study utilized the most comprehensive genome-wide association analysis of osteoarthritis to date, encompassing a meta-analysis of 13 cohorts with a total of up to 826,690 participants. By leveraging MR, this study effectively circumvented confounding factors and reverse causality. While prior research has suggested a potential link between psoriasis, psoriatic arthritis, and osteoarthritis, this study conclusively demonstrates the absence of a causal relationship between these conditions.

However, in this study, we utilized a GWAS dataset primarily based on European populations to explore the causal relationship between psoriasis and osteoarthritis. Although this provided us with high-quality information on genetic variations, we recognize that the ethnic bias inherent might limit the general applicability of our findings. For instance, specific genetic markers may vary in frequency and influence across different ethnicities, which could affect the interpretation of disease associations. For future research, we recommend incorporating data from diverse ethnic and geographical backgrounds to enhance the universality and reliability of the study results. Additionally, multicenter and cross-ethnic GWAS studies will be crucial next steps to validate our findings, ensuring the broad applicability and accuracy of the research outcomes. We explored the association between psoriasis and 4 types of osteoarthritis (OA), finding that the odds ratios (OR) exhibited wide confidence intervals (CI) and non-significant *P* values. These statistical outcomes are crucial for interpreting our findings and assessing their scientific significance. The wide confidence intervals indicate that while our analysis showed a potential association between psoriasis, psoriatic arthritis, and 4 types of OA, the precision of this association remains limited. In this study, our sample sizes were 826,690 for OA, 361,141 for psoriasis, and 361,194 for psoriatic arthritis, which may have contributed to the wide confidence intervals. We recognize sample size as a potential limitation and plan to increase the sample size in future research to enhance the stability and reliability of the results. Additionally, high variability in the data could also have broadened the confidence intervals. We used a public GWAS database and processed the data using standard 2-sample Mendelian randomization methods,^[17]^ and we have made every effort to control this variability with appropriate statistical methods. Furthermore, the non-significance of the *P* values suggests that, from a statistical standpoint, there is a lack of strong evidence for a causal relationship between psoriasis and OA.

To explore the root causes of these statistical challenges, we analyzed various factors that could affect the outcomes. First, considering the complexity of the diseases and the multifactorial influences, the association between psoriasis and OA may be affected by a combination of genetic, environmental, and lifestyle factors, making it challenging for a single GWAS analysis to capture the complete pathological mechanisms. Second, potential underrepresentation in the dataset or biases during the data collection process could also impact the results.

Based on this analysis, our study highlights the necessity of increasing sample sizes, improving data quality, and refining research designs in future work. Particularly, by expanding sample sources, enhancing the diversity and depth of research, and employing more detailed statistical methods, a more accurate understanding of the potential links between psoriasis and OA can be achieved.

#### 4.2.1. Sources of heterogeneity in Cochran Q test

We discussed the significant heterogeneity observed in the Cochran *Q* test, which may originate from the genetic backgrounds of the samples, differences in environmental factors, and variations in study design. Participants in different studies may significantly differ in terms of age, gender, ethnicity, or disease stage. Inconsistencies in data collection and measurement tools used across studies could also contribute to the heterogeneity of the results. While this heterogeneity does impact our analysis, it does not affect our overall conclusions. Future research can reduce this influence by conducting subgroup analyses to identify and analyze variables that may contribute to heterogeneity.

#### 4.2.2. Interpretation and significance of sensitivity analyses

We have added an explanation of the sensitivity analyses, including leave-one-out analysis and funnel plot analysis. These analyses demonstrate that despite potential small-scale biases, our main findings remain robust. This addition helps to underscore the reliability and applicability of our research results. The leave-one-out analysis graph shows that removing each study point has some impact on the overall effect estimate, but no single study significantly changes the direction or significance of the overall result, indicating robustness. The red line (All) represents the total effect estimate including all studies. In the funnel plot, we observe that most data points cluster near the zero effect value, although a few deviate from this area, indicating that some SNPs may slightly affect the stability of the overall MR estimate.

And in this study, we utilized a GWAS dataset that primarily focuses on the genetic markers associated with psoriasis and osteoarthritis. However, due to limitations in the data, we were unable to perform stratified analyses based on age, gender, or other demographic factors. This limitation has impacted the interpretation and generalizability of our research conclusions.

#### 4.2.3. Impact of data limitations

Stratified analysis is a crucial tool for understanding how disease risk factors vary across different population subgroups. The absence of these analyses might obscure key demographic differences that could significantly influence the genetic susceptibility and manifestation of the disease. For instance, gender and age are critical factors affecting the development of many diseases, and failing to consider these factors might mean our findings do not fully represent all populations.

#### 4.2.4. Future research directions

Given these limitations, we plan to seek and utilize datasets in future work that include more comprehensive demographic information. This will enable us to perform the necessary subgroup analyses to more accurately describe the role of genetic factors across different populations. Additionally, we will explore statistical methods to estimate and adjust for biases that may be introduced due to the lack of stratified data.

#### 4.2.5. Improvements in techniques and methods

We also plan to use multivariable adjustment models and sensitivity analyses to assess the potential impact of the absence of stratification on our research outcomes. These methods will allow us to better understand and interpret the limitations of the current results, while providing stronger data support for future research.

Increasing stratified analysis, along with enhancing the diversity and representativeness of the sample, is a key direction for our future studies. Through these efforts, we hope to provide more precise and universally applicable scientific evidence, supporting the development of prevention and treatment strategies for psoriasis and osteoarthritis.

We identified and discussed several key limitations that could impact the interpretation and application of our results.

#### 4.2.6. Residual confounding factors

Despite employing multivariable analysis to control for a variety of known confounders, the risk of residual confounding remains. Particularly, unmeasured factors related to environmental exposures and individual lifestyle choices could significantly influence our results. This type of confounding might lead to an overestimation or underestimation of the relationship strength between psoriasis and osteoarthritis.

#### 4.2.7. Exclusion of non-European populations

Our study primarily used a GWAS dataset representing European descendants. Due to genetic background differences among populations, this limits the generalizability of our findings to a globally diverse population. The genetic expressions of psoriasis and osteoarthritis may exhibit different patterns and impacts in non-European populations.

#### 4.2.8. Suggestions for future research

To overcome these limitations, we suggest that future studies should use datasets that include more comprehensive demographic information and diverse genetic backgrounds. Additionally, conducting longitudinal studies could provide deeper insights into disease progression and the mechanisms of genetic factors. These studies should be designed as multicenter, cross-ethnic to enhance the universality and accuracy of the research results.

#### 4.2.9. Enhancement of data diversity

Future research should also focus on increasing data diversity, especially by enhancing studies involving low-income countries and non-Western populations. This not only helps to fully understand the genetic basis of diseases but also ensures the broad applicability and effectiveness of scientific discoveries.

Through this comprehensive and detailed discussion, we hope not only to highlight the limitations of this study but also to demonstrate our deep understanding of and commitment to future research directions. This will provide a solid foundation for the field of genetic research into psoriasis and osteoarthritis, propelling broader and more accurate scientific exploration.

## Acknowledgments

We thank all the people who offered help with this study.

## Author contributions

**Project administration:** HaoYu Yao.

**Writing – original draft:** HaoYu Yao.

**Writing – review & editing:** HaoYu Yao.

**Supervision:** Rende Ning.

**Validation:** Rende Ning.

**Software:** Peizhi Lu, Shuo Yang, Shijie Wang.

**Formal analysis:** Ya Li, Zihao Fan.

## Supplementary Material


